# Clinical Ethics Consultation in Japan: What does it Mean to have a Functioning Ethics Consultation?

**DOI:** 10.1007/s41649-023-00257-2

**Published:** 2023-09-06

**Authors:** Noriko Nagao, Yoshiyuki Takimoto

**Affiliations:** 1 School of Nursing, Kitasato University, Sagamihara, Kanagawa Japan; 2 Department of Biomedical Ethics, Graduate School of Medicine, The University of Tokyo, Tokyo, Japan

**Keywords:** Clinical ethics consultation, Clinical ethics support system, Clinical ethics committee, Ethics committee, Mixed methods

## Abstract

This research examines the current status of clinical ethics consultation (CEC) in Japan through a nationwide study conducted with chairs of ethics committees and clinical ethics committees among 1028 post-graduate clinical teaching hospitals. We also qualitatively analyzed their viewpoints of the CEC’s benefits and problems related to hospital consultation services to identify the critical points for CEC and inform the development of a correctly functioning system. The questionnaire included structured questions about hospital CEC organization and service purpose and operation and open-ended questions about the benefits and problems of initiating CEC. The questionnaire comprised the presence/absence of an ethics committee, CEC services and membership when services were implemented, users, and the number of cases handled since inception. In addition, the respondents also provided their impressions of the CEC system’s impact on their hospital by describing (a) the benefits of CEC services and (b) the ineffectual or harmful aspects of the CEC system. Qualitative data were examined using qualitative content analysis to determine the impact of establishing a CEC and the difficulties of practice. One hundred twenty-five questionnaires were returned from either the chair of the ethics committee or clinical ethics committee in teaching hospitals. Of these, 90 (72%) reported they provided CEC services. Additionally, 36 respondents (34.6%) reported that their existing research and clinical ethics committees had conducted CEC services, and 35 (33.7%) reported having a newly established clinical ethics committee conducting CEC services. Three positive effects of establishing and four challenges in managing CEC were also identified.

## Introduction

Clinical ethics consultation (CEC), which provides support for individuals, groups, and committees for issues of conflicting values among parties involved in medical care that arise in clinical practice, is attracting attention worldwide (Aulisio 2003; Fox et al. 2007; Slowther et al. 2012). This support has existed in North America since around 1980 (McGee et al. 2002; Youngner et al. 1983). According to national surveys in the USA, all hospitals with more than 400 beds had a CEC service. Moreover, many hospitals with more than 100 beds also have CEC, and teaching hospitals reported more consultations for these services (Fox et al. 2007, 2022). This trend is due in part to the controversy over life support discontinuation for Karen Quinlan (The Supreme Court of New Jersey 1976) and the recommendation made by The Joint Commission on Accreditation of Healthcare Organizations (JCAHO) that a review system should be created by hospital ethics committees and others for addressing ethical issues that arise in clinical practice cases (Aulisio 2003). Similar support exists in Europe (Hurst et al. 2007; Reiter-Theil 2001; Slowther et al. 2012). A worldwide focus on CEC and similar support exists. In Europe and the USA, reports have been published on the approaches, roles, and activities of those who provide CEC services (Aulisio et al. 2000; Fox et al. 2007; McGee et al. 2002; Youngner et al. 1983). Healthcare professionals who consulted with CEC report that how they think about ethical issues (Orlowski et al. 2006), their educational backgrounds, and their attributes influence their susceptibility to value conflicts (DuVal et al. 2001; Hurst et al. 2007).

CEC is spreading globally (Fox et al. 2007; Fuscaldo et al. 2019; Gaudine et al. 2010; Slowther et al. 2012). Regarding whether clinical ethics consultation meets staff needs and contributes to a properly working hospital system, the number of consultations is relatively low, even in the USA (Fox et al. 2022). Ethics consultation is widespread. However, issues remain to be addressed as to whether it functions adequately.

In Japan, a 2004 survey found that 25% of hospitals designated for clinical training had CEC services (Nagao et al. 2005). In many hospitals, this task was carried out by the hospital’s ethics committee. Ethics committees within hospitals were responsible for research ethics reviews involving human subjects and advising on issues in individual cases arising in clinical practice. However, since the development of issue-based research ethics guidelines from the government after 2000, these hospital committees have placed more weight on reviewing ethical aspects of research (Akabayashi et al. 2008; Hirakawa et al. 2007).

After the 2004 survey, in 2007, the Japanese Ministry of Health, Labour, and Welfare (MHLW) issued guidelines for end-of-life medical care. Approximately 10 years have passed since these CEC guidelines on the care decision-making process were incorporated by hospitals (Ministry of Health, Labour and Welfare 2007). Thus, hospitals have been restructuring their clinical ethics support systems in recent years.

CEC services are expanding in Japan. Regarding the methods by which CEC discusses ethical issues in individual cases with consultants, Beauchamp and Childress’ (2001) four principles of biomedical ethics, developed in the USA, and the framework developed by Jonsen et al. (2002) have been widely used. Recently, however, methods developed in Europe for moral case deliberation have also been introduced (Akabayashi et al. 2008; Hattori 2019). In other cases, to address communication discord with patients and their families, mediation and support using Western CEC and medical safety methods are being developed in Japan.

Although a CEC service has been established in 73.1% of accredited hospitals in Japan, about 40% of hospitals have never had requests for CEC (Dowa et al. 2022). Moreover, challenges arise in operationalizing ethics consultation (Honke and Itai 2019). Issues exist regarding whether CEC functions effectively as a support service. To fully develop CEC in Japan, a functional system must be in place. In particular, for the system to function, its impact and barriers that prevent it from functioning must be identified and addressed.

This study evaluates the current status of CEC in Japan through administration and analysis of a nationwide study to determine the extent and effectiveness of the latest system used. We aimed to determine the current status of the mechanism: when and how often the CEC service was initiated and whether it was composed of people who provided this service. We then determined how respondents who were in a position to practice and manage CEC perceived its impact within the hospital since the initiation of services.

## Methods

We used quantitative and qualitative analytical (mixed) methods, with a self-administered questionnaire given to the chairs of ethics committees or clinical ethics committees of post-graduate clinical training hospitals across the country. All the hospitals were registered in the 2016 *Guidebook for Post-graduate Clinical Training Hospitals*. The respondent selection was sent to the chairperson of the hospital’s ethics committee. We determined that 24.7% of the hospitals designated for clinical training in the 2004 survey had an ethics committee responsible for the CEC. We used the 2004 instrument and older surveys to develop this questionnaire. A pilot study was then conducted with several CECs before distribution. The anonymous self-administered questionnaire was then mailed to the ethics committee chairs of 1028 hospitals for the full cross-sectional study.

The study was conducted from October 2016 to January 2017. The person responsible for the clinical ethics support system within each hospital or equivalent was asked to respond to the study. Respondents were asked about the structure of the CEC service, such as when and how often it was initiated and whether it consisted of people providing this service. They were also asked to freely describe the impact that the CEC service has had within the hospital.

The respondents were then asked open-ended questions in addition to structured questions about the clinical ethics support system within their hospital in a retrospective study design. The qualitative data from the free response section was then used to analyze the impact and challenges after establishing CEC.

### Data Collection

The questionnaire consists of three sections. The first section asked about the establishment of existing ethics committees and CEC. The respondents were asked about the year the ethics committee and CEC were established and their role and function using multiple-choice items and numeric responses. The questions asked included

1. Participants’ demographic data
2. Number of beds
3. The year the ethics committee was established and the role and function of the ethics committee
4. Whether or not a system for addressing ethical issues (CEC) exists, and if so, the year it was established
5. Parent organization of the CEC
6. Composition of the members of the parent organization of the CEC
7. Version of the evaluation chart when accreditation is received from the National Institute for Health Care Excellence

The item concerning the year the structure was established was added to this survey to identify trends in the development of ethics consultation since 2004 when its existence in Japan was first examined.

The second section asked about CEC activities, for example, why they started ethics consultation activities based on previous studies (DuVal et al. 2001; Fox et al. 2007; Nagao et al. 2005; Reiter-Theil 2001). Hospitals without a CEC were asked (as in 2004) about how they deal with ethical issues and what kind of system they would like to see in place. The open-ended questions asked included

1. State the reasons why you think a system for ethical issues (CEC) is necessary.
2. If there is no system for ethical issues (CEC), how are cases of ethical issues handled?

We included in our survey the number of ethics consultation providers and the number of consultations per year among hospitals designated for clinical training in Japan. This question was omitted from the 2004 national survey.

1. Number of ethical issues (CEC)
2. Characteristics of CEC providers

In the third section, the respondents were also asked to provide their impressions of the CEC system’s impact on their hospital by describing (a) the benefits of having a CEC system and (b) its ineffectual or harmful aspects.

### Analysis

#### Quantitative Analysis

Descriptive statistics were performed on the quantitative data obtained from the questionnaire. A *χ*^2^ test was performed for the presence or absence of CEC and hospital beds, classifying them according to whether they equaled or exceeded 400. The significance level was set at *p* < 0.05. SPSS version 24 was used for quantitative analysis.

#### Qualitative Analysis

All members of the research team have healthcare backgrounds and have graduate degrees in medical ethics. In addition, they have past experience conducting qualitative research. Responses directly written on self-administered questionnaires were transcribed verbatim after collection and evaluated using qualitative content analysis (Flick 2006). For qualitative content analysis, one author (NN) read document data written and named textual data using keywords (coding: safety, security, and burden supported by the CEC, difficulties in managing the CEC, and other aspects). Codes with similar meanings were gathered and classified by sub-category names (subcategorization) (NN). Then, similar subcategories were aggregated and categorized. The first author carried out the coding process with regular discussions with the coauthor (YT). Finally, the keyword and category sets were reviewed along with the initial data by the coauthor (YT). The two authors discussed any disagreements regarding the interpretation of the data and reached a consensus.

## Results

One hundred and twenty-five questionnaires were returned from teaching hospitals (response rate: 12.15%). All responses were valid. The respondents were the directors of the ethics committee or hospital ethics committee, the parent body of the hospital CEC. Table 1 displays the respondents’ characteristics. Most respondents were men in their 60s (43.2%) or 50s (38.4%). In addition, they were assistant hospital directors (44.0%) or medical doctors (21.8%). Regarding hospital characteristics, the most common type was emergency-designated hospitals (79), followed by regional medical support (60), cancer base (59), and those with specific functions (14). Hospital size was ranked in descending order: 300–399 beds (24.0%), 400–499 beds (18.4%), and 500–599 beds (14.4%) (Table 1).

**Table 1:** Demographic data

	Hospitals (*n* = 125)
Sex	
Male	113 (90.4%)
Female	7 (5.6%)
No answer	5 (4.0%)
Age	
30s	4 (3.2%)
40s	11 (8.8%)
50s	48 (38.4%)
60s	54 (43.2%)
70s	3 (2.4%)
No answer	5 (4.0%)
Position	
Assistant director of the hospital	55 (44.0%)
Medical doctor	28 (21.8%)
Chair of the hospital	23 (22.4%)
Administrator	5 (4.0%)
Other	14 (11.2%)
No answer	
Hospital characteristics (multiple responses)	
Advanced treatment hospital	14
Regional medical support hospital	60
Emergency hospital	79
Hospital for cancer treatment	59
Hospital with special disease care units	17
Others	5
Beds	
Less than 200 beds	5 (4.0%)
200–299 beds	14 (11.2%)
300–399 beds	30 (24.0%)
400–499 beds	23 (18.4%)
500–599 beds	18 (14.4%)
600–699 beds	16 (12.8%)
700–799 beds	9 (7.2%)
800–899 beds	4 (3.2%)
900–999 beds	1 (0.8%)
More than 1000 beds	5 (4.0%)

### Current Structure and Trends in CEC

The respondents were asked about the presence/absence of CEC services in 2016. Ninety (72%) respondents from teaching hospitals reported that they had the service. The questionnaire asked how the hospital provided CEC. Thirty-six (34.6%) respondents answered that the existing research and clinical ethics committees had conducted CEC. Thirty-five (33.7%) respondents answered that the newly established clinical ethics committee had conducted it (Table 2).

**Table 2:** Mechanism of clinical ethics consultation (CEC) present at hospital

Clinical ethics committee	35 (33.7%)
Ethics committees (both research ethics and clinical ethics)	36 (34.6%)
Special branch of clinical ethics	13 (12.5%)
Committee coping with specific issues	6 (6%)
Clinical ethics task force in nursing administration	6 (6%)
Others	8 (7.7%)
(Total answers = 104)	

**Fig. 1:**
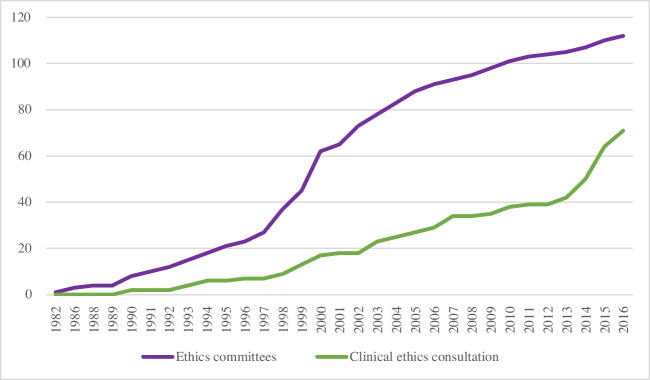
Annual change of ethics committees and clinical ethics consultation

Respondents were also asked about the timeline for the provision of CEC services. Their answers revealed that CEC was initially provided by an ethics committee in 1990, after which its provision by ethics committees gradually expanded. Concerning independent clinical ethics committees acting as CEC providers, 1993 was reported as the earliest occasion, followed by an increasing number of similar instances in 2002, 2006, and 2014 (Fig. 1). Until 2004, 25 hospitals had a new CEC mechanism; from 2005 to 2010, 14 hospitals had a new CEC mechanism. From 2011 to 2016, 34 hospitals indicated that they had started a new CEC mechanism.

We divided hospitals into two groups based on the number of beds, fewer than 400 and 400 or more. No significant relationship was found between the size of hospitals and whether CEC was provided (Table 3). Respondents were also asked how many times consultation was requested. Fifty-six (70%) reported 1–10 requests a year. Seven (8.8%) respondents reported having none since they started the CEC service. A total of 21.3% of respondents received requests for more than 12 consultations annually.

**Table 3:** Presence/absence of CEC services and number of beds

	2016	Beds		Total	χ^2^ test
		< 400 beds	≧ 400 beds		*p* value
Presence/absence of CEC services	Presence	33	57	90	*p* = 0.233
	Absence	16	19	35	
		49	76	125	

Respondents were asked if they had received a hospital functionality evaluation accreditation from the Agency for Evaluations of Hospital Management and Health Care Provision and (if so) which version was administered. Fifty-seven hospitals (56%) were most likely to have received the 3rd generation version^1^, followed by 26 respondents who received the 6th version^2^ and four respondents who received the 5th version^3^ (Table 4). A statistically significant difference was found when comparing the presence or absence of CEC, with or without accreditation from the Japan Council for Quality Health Care (*p* < 0.002) (Table 5).

**Table 4:** Hospitals with and without accreditation from the Japan Council for Quality Health Care

With accreditation		100
	Version of evaluation form	
	Ver. 2	1 (1%)
	Ver. 3	1 (1%)
	Ver. 5	4 (4%)
	Ver. 6	26 (26%)
	3G	57 (56%)
	no answer	11 (11%)
Without accreditation		13

**Table 5:** Presence/absence of CEC services and with/without accreditation from the Japan Council for Quality Health Care

		With or without accreditation from Japan Council for Quality Health Care		χ^2^ test
				*p* value
		With accreditation	Without accreditation	
Presence/absence of CEC services	Presence	76	4	0.002
	Absence	24	9	

Table 6 lists CEC members. Most members were medical doctors, some with ethics education, such as short-term intensive seminars and workshops, and others without it. Other members included nursing managers, administrative staff, other healthcare professionals (pharmacologists, physical and occupational therapists, dieticians, and others), legal scholars or lawyers, certified nursing specialists, and nurses who had received ethics education.

**Table 6:** HEC members

Doctor with ethics education	56
Doctor without ethics education	61
Nursing service manager	75
Specialized/certified nurse	12
Nurse with ethics education	12
Other healthcare providers	38
Administrative staff	44
Jurist/lawyer	29
Philosopher/ethicist	8
Other	25
	(TA = 360)

### Qualitative Results

A qualitative content analysis of the responses to the open-ended questions obtained from 83 respondents who indicated that they had CEC was performed. Fifty-six responses described a positive impact, and 27 recounted difficulties that arose after establishing CEC. This procedure resulted in the identification of 75 codes and 23 subcategories related to three positive impact categories resulting from the establishment of a CEC system (Table 7). In addition, 26 codes, 12 subcategories, and four categories were extracted for problems after the establishment of the CEC (Table 8). Categories are indicated by “ ” and subcategories are indicated by ‘ ’.

**Table 7:** Positive effects of the establishment of the ethics consultation

Category	Sub-category
Feel safe and secure by having gone through an organized procedure	Safety and security are guaranteed by having gone through an ethics committee
	Medical care can be provided based on multidisciplinary and comprehensive judgment
	The burden and suffering of doctors are reduced
	The psychological burden and suffering of hospital staff are reduced
Provided a mechanism for hospitals to support policies and responses based on legal and ethical considerations	The hospital’s support for discussion
	The hospital’s opportunity to discuss policies and responses
	The ability to obtain legal advice and resolve issues
	The hospital’s ability to advise medical decisions based on ethical considerations as a third party
The ethics support system improves staff awareness and control of medical care in the hospital	Supporting the relationship between hospital staff and patients
	Improving staff awareness of ethics
	Enabling a quick response to urgent issues
	Enabling the hospital to control issues and direction within the hospital
	Generating interest in ethics support and establishing a network

**Table 8:** Problems in managing ethics consultation

Category	Sub-category
Difficulty in coordinating time for CEC	Difficulty in expediting the process by including outside committee members
	Difficulty in scheduling committee meetings when responding quickly
	Difficulty in securing the time of the person in charge when responding quickly to internal questions
Problems related to cases handled by CEC	Difficulties in judgment and response by CEC
	Difficulties in drawing a line between issues to be discussed by the Ethics Committee and issues to be discussed by the Ethics Committee
	Difficulties in raising cases that should be discussed by ethics consultation
Misunderstanding of the function of CEC	Demanding judgment from the hospital and leaving it to the hospital
	Confusion of functions and roles with other hospital organizations (on the part of the medical staff)
Immaturity of the CEC system	Lack of a system for CEC
	Lack of a dedicated person in charge of CEC
	Lack of guaranteed objectivity by not including an outside committee member

Three constructive effects of establishing CEC were identified. The first was the impact on medical care providers, who were able to “feel safe and secure by having gone through an organized procedure,” which consisted of ‘safety and security are guaranteed by having gone through an ethics committee,’ ‘medical care can be provided based on multidisciplinary and comprehensive judgment,’ ‘the burden and suffering of doctors are reduced,’ and ‘the psychological burden and suffering of hospital staff are reduced.’ The second was the positive impact of the CEC system. It “provided a mechanism for hospitals to support policies and responses based on legal and ethical considerations,” which consisted of ‘the hospital’s support for discussion,’ ‘the hospital’s opportunity to discuss policies and responses,’ ‘the ability to obtain legal advice and resolve issues,’ and ‘the hospital’s ability to advise medical decisions based on ethical considerations as a third party.’

Finally, the third positive change was that “the ethics support system improves staff awareness and control of medical care in the hospital,” consisting of ‘supporting the relationship between hospital staff and patients,’ ‘improving staff awareness of ethics,’ ‘enabling a quick response to urgent issues,’ ‘enabling the hospital to control issues and direction within the hospital,’ and ‘generating interest in ethics support and establishing a network.’

Four problems in managing CEC were also identified. The first was “difficulty in coordinating time for CEC,” consisting of ‘difficulty in expediting the process by including outside committee members,’ ‘difficulty in scheduling committee meetings when responding quickly,’ and ‘difficulty in securing the time of the person in charge when responding quickly to internal questions.’ The second was “problems related to cases handled by CEC,” consisting of ‘difficulties in judgment and response by CEC,’ ‘difficulties in drawing a line between issues that the ethics committee discussed anyway because healthcare providers met trouble and ethical issues that should have been discussed by the ethics committee,’ and ‘difficulties in raising cases that should be discussed by CEC.’ The third was “misunderstanding of the function of CEC,” consisting of ‘demanding judgment from the hospital and leaving it to the hospital’ and ‘confusion of functions and roles with other hospital organizations (on the part of the medical staff).’ The fourth was “immaturity of the CEC system” on the part of those responsible for ethics consultation for medical care providers in terms of operational issues consisting of ‘lack of a system for CEC,’ ‘lack of a dedicated person in charge of CEC,’ and ‘lack of objectivity guaranteed by not including an outside committee member.’

## Discussion

This survey reveals the latest status of CEC, an ethics support in clinical practice in Japan. It found that in 2004, about 25% of hospitals designated for clinical training had CECs. In 2016, 71.6% had this system. Furthermore, CEC was a function of existing ethics committees and newly established clinical ethics committees. Until 2013, 1–3 hospitals had CECs per year. Since then, the number of hospitals with CECs has increased to eight in 2014 and 14 in 2015. The presence of CEC was not significantly different between hospitals with more than 400 beds and those with fewer, indicating that hospitals had this mechanism regardless of their size. However, the largest number of requests for CEC was 1–10 per year, with some respondents reporting no requests. This finding indicates that although CEC has been established in Japan, it is not yet fully functional. This section discusses our results in relation to the background that has influenced CEC development.

An external factor may have led hospitals designated for clinical training to develop CEC systems, that is, the accreditation of hospital functionality evaluation. The Japan Council for Quality Health Care (JCQH) started evaluating hospital functions in 1996 to assess medical care’s quality and safety. Since then, JCQH has revised the evaluation items every 5 years. Each time, the items related to clinical ethics have been revised to evaluate the actual performance of the hospital, from the establishment of a clinical ethics system to how it manages ethical issues and the hospital’s understanding of them. The present study’s results indicate that the number of teaching hospitals that established CEC services increased in 2007 and 2015 in response to these revisions. The external evaluation of hospitals may motivate the establishment and organization of new systems, such as CEC. However, this survey reports numbers of consultations ranging from 1–10. Some hospitals have had no consultations since their inception. Therefore, although the mechanisms have been established, they may not function properly. As the results of the analysis indicate, the reasons for the lack of functionality include issues related to the operation of the CEC system, such as “difficulty in coordinating CEC time,” “problems related to cases handled by the CEC system,” and “immaturity of the CEC system” in which the person in charge is not identified. These issues were identified in the operation of the CEC system. In addition, issues arise on the consulting side, such as “misunderstanding of the function of CEC on the part of medical professionals.”

One solution to the problems associated with CEC operation, such as the need to ensure coordination of dates and times, the speed of response, and the immaturity of the consultation system, is to appoint a full-time person to supervise and centralize the contact points. However, even in the USA, a leading CEC country, few professionals have formal CEC education. Furthermore, difficulties have been reported in assigning a full-time person to this task due to operational obstacles. CEC is reported to be conducted by teams with various educational backgrounds (Fox et al. 2007). In Japan, where CEC has just started, assigning full-time ethics consultants is challenging, although training of ethics support personnel has begun. In such a situation, CEC might be operated in Japan within a banking system that pools roughly 20 people who can provide consultation and respond to problems as they arise. However, educating supervisors on the CEC knowledge and skills required is necessary. Brief educational programs are available for the education of ethics consultants in Japan. In addition, graduate education programs have begun to provide the knowledge necessary for CEC. However, compared to the USA, education and certification in Japan are still in their infancy. Currently, a need exists to build the knowledge and skills required for those who practice CEC to resolve ethical issues in clinical practice in Japan. At this point in time, when the certification of ethics consultants is still in its early stages, one alternative solution is peer education, in which those who work together collaborate and build on each other’s knowledge and skills. In Japan, the Hospital and Clinical Ethics Committee Collaboration Conference was launched in 2019. Efforts to share clinical ethics support activities among hospitals with specific functions began voluntarily (Takeshita et al. 2022). In addition, the Clinical Ethics Consortium was launched to create a forum for those responsible for CEC in clinical settings to collaborate, exchange information, and discuss issues. The accumulation of opinions and discussions on CEC activities at each facility in the future will help improve professional knowledge and skills.

Furthermore, “misunderstanding of the function of CEC” was identified as a problem for those who requested a CEC. The first problem, “seeking the hospital’s judgment and throwing the decision to the hospital,” may be a mismatch between the expectations of the consulting healthcare professionals for CEC and ethics consulting practitioner functions. Value conflicts that arise in clinical practice require adjustments and compromises that are acceptable to both parties. However, some healthcare professionals are frequently exhausted from conflicts with patients and their families. They would prefer that the CEC decide. Especially in Japan, the discontinuation of life support equipment is detached from any legal basis for the patient’s advance directive, in contrast with Europe and the USA. In some cases, the risk of criminal prosecution is considered to be a psychological defense, promoting feelings of exhaustion and emotion. A change in mindset is needed to address these problems. Discussing the issue from a domestic legal and ethical perspective may also protect healthcare professionals. Furthermore, opportunities to introduce support and education about ethical considerations are needed.

The other problem is that of ‘confusion of functions and roles’ with other hospital organizations. The problem may stem from steps taken by hospitals to increase the number of CEC requests, as inferred from their scarce number. Hospitals consult with frontline healthcare providers about managing medical care challenges and difficult cases. Moreover, they also consult with patients about difficult cases when responding to their complaints. Of course, patient complaints may also include ethical issues requiring assistance, such as that offered by CEC. However, given the current state of CEC in Japan, providing a wide range of consultation services, including various types of consultation, is required to make the CEC system function. However, some people may be uncertain about what they need, so they may decide to seek advice in any case, which may lead to problems in making distinctions.

In addition to the challenges of CEC, the survey also revealed the constructive impact of this service. In particular, our findings indicate that they “feel safe and secure by having gone through an organized procedure” and that it “provided a mechanism for hospitals to support policies and responses based on legal, ethical considerations.” Requests for CEC are often accompanied by a value conflict between the healthcare professional and the patient/family, resulting in emotional communication, an inability to dialog, and a relationship in which trust is undermined. Therefore, the presence of an ethics consultant as an objective party to the case and the parties involved is intended to neutralize the situation (Stephens et al. 2019). In addition, a positive impact was extracted from the fact that “the ethics support system improves staff awareness and control of medical care in the hospital.” Consulting a CEC can lead to reassurance in resolving conflicts over medical care with patients and their families and confidence in basing the medical care options being contemplated on ethical considerations. Moreover, it is beneficial for managing medical care within the hospital and helping to ensure its appropriateness and fairness (Fox et al. 2022; Hauschildt and De Vries 2020).

## Limitations

This study focused exclusively on teaching hospitals. Because the response rate was relatively low, only hospitals with a strong interest in clinical ethics support structures may have responded. Consequently, the results may not represent all Japanese teaching hospitals. Furthermore, while this survey targeted teaching hospitals, this type comprises only 13.9% of all hospitals in Japan (1028/7379 as of December 2016).

Respondents from hospitals with CEC were engaged in the practice. However, some staff had received various forms of education in clinical ethics, short intensive courses, and graduate courses, while others had not. Additionally, respondents from hospitals without CEC were not engaged in it. Which clinical ethics consultants had a master’s or doctoral degree in ethics also remains unclear. Some staff likely had not received any education in clinical ethics. Thus, responses were diverse and cannot be generalized.

After this study, the COVID-19 pandemic occurred. Many hospitals faced complex problems involving decision-making and resource allocation for life-sustaining treatment. Therefore, the number of hospitals with new CECs may have increased. Hence, the current status of CEC systems in all Japanese hospitals deserves further study. As this study is an overview of current CEC mechanisms and their activities, outcome assessment and quality assurance require future investigation. Only a small number of medical institutions answered, and those who did were interested in CEC. Therefore findings obtained in this study may not be typical for clinical training hospitals in Japan. The number and type of hospitals answered in 2004 and 2016 differed. However, these studies are the first to provide insight into changes in CEC services over the last decade in Japan.

Because respondents to the semi-structured questions were active in the development and activities of CEC, their answers cannot be generalized. However, they objectively described the positive impacts and difficulties associated with the establishment of CEC at each facility. Promising future research includes conducting a qualitative study of those involved in CEC to clarify the process of organizing the service and the skills required for responsible parties.

## Conclusions

This study determined the current status of CEC using a nationwide survey to determine the extent of its use in Japan. Many hospitals in Japan have an internal hospital CEC system. However, the number of consultations per year varies. Moreover, the system functions only intermittently.

The difficulties with CEC identified in this study are important issues that need to be addressed for effectual CEC. These issues include difficulty scheduling requests for ethics support, uncertainty about what kind of consultation should be provided, and misunderstanding of its functions. However, the results of the Japanese survey may help promote CEC in societies with similar social backgrounds, such as those with feudalistic medical systems, physician authority, and family-centeredness. In the context of family relationships and paternalistic society, we believe that healthcare providers are likely to face strong ethical conflicts, such as the inability to provide patient-centered medical care and to respect patients’ rights, because these rights are obscured by family members’ claims. Therefore, CEC is expected to play a role in promoting respect for patients’ rights between medical professionals and their families, facilitating dialog in the service of practicing good patient care, and managing conflicts between medical professionals and their families. Also, in a society where physicians have strong authority, other professions may avoid dialog with them. At such times, CEC may play a role in alleviating the distress that these other medical professionals may have. We believe that the Japanese way of doing CEC, with a multidisciplinary team, is one promising model.

## Data Availability

The data that support the findings of this study are available from the corresponding author, (NN), upon reasonable request. All data are in Japanese.

## Footnotes

1 Japan Council for Quality Health Care. 2013. The Evaluation of Hospital Management and Health Care Provision. The 3rd generation. In the “Clinical Ethics” section, a hospital-wide mechanism for reviewing clinical ethics issues existed, and ongoing efforts were made to resolve these.

2 Japan Council for Quality Health Care. 2009. The Evaluation of Hospital Management and Health Care Provision. The 6th version. In the “Clinical Ethics” section, hospital-wide clinical ethics issues are identified and reviewed.

3 Japan Council for Quality Health Care. 2005. The Evaluation of Hospital Management and Health Care Provision. The 5th version. In the “Clinical Ethics” section, clinical ethics issues are identified and reviewed.

## Abbreviations

CEC: clinical ethics consultation
JCQH: Japan Council for Quality Health Care
